# Long COVID: persistent impacts and challenges for speech-language-hearing therapy

**DOI:** 10.1590/2317-1782/e20240291en

**Published:** 2025-10-13

**Authors:** Aline de Souza Silva, Rodrigo Dornelas, José Roberto Lapa e Silva

**Affiliations:** 1 Faculdade de Medicina, Universidade Federal do Rio de Janeiro – UFRJ - Rio de Janeiro (RJ), Brasil.

Dear editors,

Since the outbreak of the COVID-19 (coronavirus disease 2019) pandemic, experts have continuously investigated its consequences. Some studies indicate that symptoms of the disease are not resolved with recovery from the infection; many people who were diagnosed with COVID-19 continue to suffer lingering effects^(1-3)^. The World Health Organization (WHO) estimates that 10% to 20% of those who recovered from COVID-19 experience prolonged symptoms. The prevalence ranges from 10-30% among non-hospitalized patients to 50-70% among hospitalized patients and is lower (10-12%) in the vaccinated population^(4,5)^. Studies show that vaccination reduces the prevalence of long COVID by 20.9% in the United States and 15.7% worldwide^(4,6)^. The WHO defines these symptoms as long COVID or post-COVID condition when they arise up to 3 months after infection, persist for at least 2 months, and cannot be explained by other diagnoses^(7-9)^.

The G20 (Group of 20) is an international forum comprising the countries with the world’s largest economies. It currently plays a central role in coordinating economic policies with global impact, aiming, at least rhetorically, to promote inclusive growth, reduce inequalities, and respond to global challenges. In 2024, Brazil assumed the chair of the G20^(10)^. The third meeting of the G20 Health Working Group in Rio de Janeiro (June 2024) addressed the impacts of COVID-19 as a global public health issue. On the occasion, the WHO also highlighted the need for a precise definition for long COVID to improve communication between health professionals and the population, facilitating care and the development of treatments^(11)^.

Long COVID poses a significant challenge to public health and the economy, increasing costs, disability, and lost productivity. Addressing this challenge requires coordinated approaches to meet the patients’ various needs, ranging from diagnosis and treatment to social and economic support, with attention to the long-term effects on health and functional performance. The Pan American Health Organization (PAHO) suggests that the G20 adopt strategies to manage long COVID, including increased investment in multidisciplinary research, effective scientific communication methods, and better organization of health systems to access therapeutic interventions^(11)^.

This condition can affect people of all ages, but is more common in women, those with type 2 diabetes, aged 65 years or more, with pre-existing comorbidities, vulnerable groups, with lower education levels, or who had severe forms of the disease and were admitted to intensive care units)^(2,5,12)^. However, more than 30% of those affected have no pre-existing conditions^(4)^. Most cases occur in non-hospitalized people with mild disease, who account for most COVID-19 cases worldwide^(4)^.

Long COVID can affect several organs and systems, with more than 200 reported signs and symptoms, including chronic fatigue syndrome, dyspnea, physical and cognitive problems such as memory and concentration loss, headache, cardiovascular disease, cough, loss of smell and taste, and dysautonomia. They can persist, vary, and range from mild to severe, requiring a multidisciplinary treatment approach^(4,7,8,13,14)^.

Speech-language-hearing pathologists play an important role in managing difficulties resulting from long COVID, covering speech, language, communication, swallowing, and voice. Their work contributes to the patients' functional rehabilitation and quality-of-life improvement. However, despite advances in understanding this condition, there is still little speech-language-hearing research dedicated to the topic. Most studies focus on hospitalized patients who needed intensive care, mechanical ventilation, intubation, or tracheostomy weaning^(15-20)^. There are significant reports of speech-language-hearing follow-up for dysphonia (17.1-37.0%) and dysphagia (7.8-27.0%) in the post-acute phase of COVID-19 after hospital discharge^(15,18)^. While some studies indicate that swallowing function may be almost normal at hospital discharge^(17,19)^, others reveal persistent difficulties with voice and swallowing and changes in the upper airways, reinforcing the need for more comprehensive investigations and specialized interventions^(20-22)^.

Thus, there is a significant gap in studies focused on speech-language-hearing care needs for the non-hospitalized population. This is relevant because speech-language-hearing services receive referrals for individuals with long COVID, presenting symptoms such as dysphonia, dysphagia, respiratory disorders, and chronic cough^(23)^.

Currently, there is limited evidence assessing voice, swallowing, and communication difficulties in individuals with long COVID^(24)^. Furthermore, there is a paucity of detailed information on how multidisciplinary healthcare is delivered for long COVID. Further studies are needed to improve support for individuals with long COVID, increase access to information on relevant symptoms, highlight the role of speech-language-hearing therapy in the rehabilitation of voice, swallowing, and communication difficulties, and influence national guidelines for resources and an appropriate multidisciplinary approach for this complex condition.
