# Supplementary material for: Long COVID: persistent impacts and challenges for speech-language-hearing therapy
Source: Codas. 2025 Oct 13;37(5):e20240291. doi: 10.1590/2317-1782/e20240291en (PMC12533526; doi:10.1590/2317-1782/e20240291en)
Supplement: Translation

# Translation: COVID Longa: impactos persistentes e os desafios para a Fonoaudiologia

**DOI:** 10.1590/2317-1782/e20240291pt

**Authors:** Aline de Souza Silva, Rodrigo Dornelas, José Roberto Lapa e Silva

**Affiliations:** 1 Faculdade de Medicina, Universidade Federal do Rio de Janeiro – UFRJ - Rio de Janeiro (RJ), Brasil.

Caros, editores

Desde o início da pandemia de COVID-19 (Coronavirus Disease 2019), especialistas continuam a investigar as implicações dessa doença. Alguns estudos indicam que os sintomas da doença não cessam com a recuperação da infecção; muitas pessoas que tiveram o diagnóstico de COVID-19 continuam sofrendo efeitos persistentes^(1-3)^. A Organização Mundial da Saúde (OMS) estima que 10% a 20% dos recuperados de COVID-19 enfrentam sintomas prolongados. A prevalência varia de 10-30% entre não hospitalizados e de 50-70% entre hospitalizados, sendo menor (10-12%) na população vacinada^(4,5)^. Estudos mostram que a vacinação reduz a prevalência de COVID Longa em 20,9% nos Estados Unidos e 15,7% globalmente^(4,6)^. A OMS define esses sintomas como “COVID Longa” ou “Condição Pós-COVID” quando surgem até três meses após a infecção, persistem por pelo menos dois meses e não podem ser explicados por outros diagnósticos^(7-9)^.

O G20 (Grupo dos Vinte) é um fórum internacional composto pelos países com as maiores economias do mundo, que atualmente tem centralidade na coordenação das políticas econômicas com impacto global, com objetivo, ao menos retórico, de promoção do crescimento inclusivo, redução das desigualdades e resposta aos desafios globais. Em 2024, o Brasil assumiu a presidência do G20^.(10)^ Na terceira reunião do Grupo de Trabalho de Saúde do G20 no Rio de Janeiro (Junho, 2024), foi abordado os impactos da COVID-19 como uma questão de saúde pública global. Neste espaço, a OMS destacou ainda a necessidade de uma definição precisa para a COVID longa com o objetivo de melhorar a comunicação entre profissionais de saúde e a população, facilitando o atendimento e o desenvolvimento de tratamentos^(11)^.

A COVID longa representa um desafio significativo para a saúde pública e a economia, aumentando custos, incapacidade e perda de produtividade. Enfrentar esse desafio exige a implementação de abordagens coordenadas que atendam às diversas demandas dos pacientes, abrangendo desde diagnóstico e tratamento até suporte social e econômico, com atenção aos efeitos prolongados na saúde e no desempenho funcional. A Organização Pan-Americana da Saúde (OPAS) sugere que o G20 adote estratégias para gerenciar a COVID longa, incluindo mais investimentos em pesquisas multidisciplinares, métodos eficazes de comunicação científica e melhor organização dos sistemas de saúde para acesso a intervenções terapêuticas^(11)^.

Essa condição pode afetar pessoas de todas as idades, porém é mais comum em adultos do gênero feminino, com diabetes tipo 2, idade acima de 65 anos, comorbidades preexistentes, grupos vulneráveis, menor nível educacional, ou que tiveram formas graves da doença e foram internadas em UTIs (Unidades de Terapia Intensiva)^(2,5,12)^. No entanto, mais de 30% dos afetados não têm condições preexistentes^(4)^. A maioria dos casos ocorre em pessoas não hospitalizadas com grau leve da doença, que representam a maior parte dos casos de COVID-19 globalmente^(4)^.

A COVID Longa pode afetar diversos órgãos e sistemas, com mais de 200 sinais e sintomas relatados, sendo alguns deles: síndrome da fadiga crônica, dispneia, problemas físicos e cognitivos como perda de memória e concentração, cefaléia, doenças cardiovasculares, tosse, perda de olfato e paladar, e disautonomia, que podem variar de leves a graves e, persistir ou variar, exigindo uma abordagem multidisciplinar para o tratamento^(4,7,8,13,14)^.

Os fonoaudiólogos desempenham um papel importante no manejo das dificuldades decorrentes da COVID longa, abrangendo fala, linguagem, comunicação, deglutição e voz. Sua atuação contribui na reabilitação funcional e para a melhoria da qualidade de vida dos pacientes, mas, apesar dos avanços na compreensão dessa condição, ainda há poucas pesquisas fonoaudiológicas dedicadas ao tema. A maioria dos estudos concentra-se em pacientes hospitalizados que necessitaram de cuidados intensivos, ventilação mecânica, intubação ou desmame de traqueostomia^(15-20)^. Após a alta hospitalar, há relatos significativos de acompanhamento fonoaudiológico para disfonia (17,1-37,0%) e disfagia (7,8-27,0%) na fase pós-aguda da COVID-19^(15,18)^. Enquanto algumas pesquisas indicam que a função de deglutição pode estar quase normal na alta hospitalar^(17,19)^, outras revelam dificuldades persistentes de voz, deglutição e alterações nas vias aéreas superiores, reforçando a necessidade de investigações mais abrangentes e intervenções especializadas^(20-22)^.

Assim, há uma lacuna significativa em estudos focados nas necessidades de atendimento fonoaudiológico para a população não hospitalizada. Isso é relevante porque os serviços fonoaudiológicos estão recebendo encaminhamentos para indivíduos com COVID Longa, apresentando sintomas como disfonia, disfagia, distúrbios respiratórios e tosse crônica^(23)^.

Atualmente, existem poucas evidências que avaliem as dificuldades de voz, deglutição e comunicação em indivíduos com COVID Longa^(24)^. Além disso, há uma escassez de informações detalhadas sobre como os serviços são prestados no contexto de cuidados multidisciplinares para a COVID Longa. É importante que se tenha estudos para melhorar o suporte aos indivíduos portadores da COVID Longa, aumentar o acesso à informação sobre os sintomas relevantes, destacar o papel da Fonoaudiologia na reabilitação de dificuldades de voz, deglutição e comunicação, e influenciar as diretrizes nacionais para uma abordagem multidisciplinar adequada e recursos apropriados para essa condição complexa.
